# Perioperative Treatment of Brain Arteriovenous Malformations Between 2006 and 2014: The Helsinki Protocol

**DOI:** 10.1007/s12028-019-00674-y

**Published:** 2019-02-14

**Authors:** Tarmo Niini, Aki Laakso, Päivi Tanskanen, Mika Niemelä, Teemu Luostarinen

**Affiliations:** 1 Division of Anesthesiology, Department of Anesthesiology, Intensive Care and Pain Medicine, University of Helsinki and Helsinki University Hospital, Helsinki, Finland; 2 Department of Neurosurgery, University of Helsinki and Helsinki University Hospital, Helsinki, Finland

**Keywords:** Intracranial arteriovenous malformations, Neurosurgical intensive care unit, Postoperative hemorrhage, Retrospective study, Blood pressure, Clinical protocols

## Abstract

**Objective:**

We reviewed retrospectively the perioperative treatment of microsurgically resected brain arteriovenous malformations (bAVMs) at the neurosurgical department of Helsinki University Hospital between the years 2006 and 2014. We examined the performance of the treatment protocol and the incidence of delayed postoperative hemorrhage (DPH).

**Methods:**

The Helsinki protocol for postoperative treatment of bAVMs was used for the whole patient cohort of 121. The patients who had subsequent DPH were reviewed in more detail.

**Results:**

Five out of 121 (4.1%) patients had DPH. These patients had a higher Spetzler–Martin grade (SMG) (*p* = 0.043) and a more complex venous drainage pattern (*p* = 0.003) as compared to those who had no postoperative bleed. Patients with DPH had 43% larger intravenous fluid intake in the neurosurgical intensive care unit (*p* = 0.052); they were all male (*p* = 0.040) and had longer stay in the intensive care unit (*p* = 0.022).

**Conclusions:**

The Helsinki protocol for postoperative treatment of bAVMs was found to produce comparable results to a more complex treatment algorithm. DPH was associated with high SMG, complex venous drainage pattern, male gender and high intravenous fluid intake. Our findings support the use of SMG in defining patient’s postoperative treatment as the DPHs in our study occurred in patients with grade 2–5.

## Introduction

Brain arteriovenous malformations (bAVMs) are a known cause of intracranial hemorrhage. BAVMs are found either incidentally (2–10%) or due to symptomatic presentation. BAVM may present with headache (6–14%), symptomatic epilepsy (18–35%) or a focal neurological deficit (3–10%). However, hemorrhagic stroke is the most common presentation for a bAVM (45–72%) [1]. Microsurgical resection is the current treatment option for bAVMs, alongside with observation, radiosurgery or endovascular treatment. Microsurgery is indicated if the operation is technically feasible, and the risks at an acceptable level [2–4]. The most feared complication of bAVM surgery is delayed postoperative hemorrhage (DPH). The incidence of DPH ranges from 2 to 21% depending on the study [5–11]. The Spetzler–Martin grading (SMG) system is used to evaluate the risk of surgical treatment of bAVMs. Grading is based on the size of the nidus, the eloquence of adjacent brain and the venous drainage of the nidus, with higher grade indicating greater risk for complications, such as DPH [12].

The lack of a normal capillary network creates a shunt allowing abnormal blood flow from the arterial side to the venous side. This leads to a high-pressure environment in the venous side with risk of hemorrhage, if the vein tears. Redistribution of the blood flow following the removal of the bAVM may cause the surrounding brain parenchyma to swell and increase the risk of DPH. This may be explained with the normal perfusion pressure breakthrough hypothesis [13]. However, the underlying pathophysiology of edema and hemorrhage after bAVM resection remains controversial. Tight postoperative blood pressure control and a restrictive intravenous fluid management regime are used to decrease the risk of DPH [14–17].

Successful surgical treatment of bAVMs requires good microsurgical skills and anesthesiologic expertise. The basic treatment principles regarding microsurgery and neuroanesthesia at our department have been published previously. These guidelines aim at simple and fast operations preserving normal anatomy and maintaining stable hemodynamics while securing adequate cerebral perfusion [18, 19]. The aim of this study was to investigate the incidence of DPH following surgical excision of bAVMs when the Helsinki protocol for the perioperative treatment was used and to compare it to a comparable, albeit more complex, treatment protocol published by Morgan et al. [11].

## Materials and Methods

After approval from Helsinki University Hospital Scientific and Ethical Board, we retrospectively reviewed the hospital records of the patients who had undergone surgery for bAVM in the neurosurgical department of Helsinki University Hospital between the years 2006 and 2014. For this type of study, formal consent is not required. We collected data from the Helsinki AVM database, surgical and intensive care unit database and patient medical records.

The collected variables included age, sex, bAVM size and location, SMG, preoperative embolization, treatment related data during neurointensive care unit (NICU) stay (daily target systolic blood pressure [SBP], daily achieved SBP, daily fluid intake and output from the first postoperative day (POD) onward, daily maximum and minimum central venous pressure, length of time spent in ventilator), length of NICU stay and neurological outcome at 2–4 months from surgery and incidence of DPH. Daily SBP target was achieved if SBP did not exceed the limit at all during the day. The Glasgow Outcome Scale (GOS) was determined by the treating neurosurgeon at the first follow-up visit and retrieved from the patient medical records.

DPH was defined as brain hemorrhage into the resection bed or brain parenchyma that was not evident in postoperative imaging and resulted in a need for reoperation or a new neurological deficit, which occurred prior to discharge from the hospital. The same definition was used by Morgan et al. in their study [11].

### Helsinki Protocol for Postoperative Treatment of bAVMs

All patients at the neurosurgical department of Helsinki University hospital treated for bAVMs follow the same postoperative treatment protocol according to the SMG. We describe the protocol in the following paragraphs and in Table 1.

**Table 1:** Treatment guidelines by protocol group

Group I	Group II	Group III
Patients with small, unruptured bAVMs of Spetzler–Martin grade 1 without surgical complications No need for a central venous catheter Extubation in the operating room and follow-up in NICU Normal blood pressure (SBP generally under 150 mmHg) Strict fluid balance postoperatively Antihypertensive medication if needed (vasodilating beta blocker or a calcium channel blocker) for 1 to 2 weeks postoperatively	Patients with medium to large bAVMs without and small bAVMs with surgical complications or if ruptured preoperatively Recovery from general anesthesia in NICU. According to the clinical evaluation, propofol/dexmedetomidine sedation until the postoperative control imaging (CT-scan, DS- or CT-angiogram) has been performed. Head of bed elevation 15–30% Restricted intravenous fluid administration postoperatively Low SBP target (generally under 120–130 mmHg) and avoidance of sudden increase in SBP Labetalol, hydralazine and clonidine are used to lower blood pressure Dexmedetomidine for sedation (0.2–1.5 µg/kg/h infusion) Patient in NICU until stabilized, usually up to several days Antihypertensive medication (a vasodilating beta blocker or a calcium channel blocker) for 1 to 2 weeks postoperatively	Patients with large bAVMs Placement of central venous catheter preoperatively Propofol and/or dexmedetomidine sedation until the postoperative control imaging has been performed or even longer Head of bed elevation 15–30% Restricted intravenous fluid administration postoperatively Avoidance of any sudden increase in SBP Low SBP target: first under 90–110 mmHg, then gradually increased 110 → 120 → 130 → 140 → 150 mmHg Patient in NICU until stabilized, usually up to several days Antihypertensive medication (a vasodilating beta blocker or a calcium channel blocker) for one to two weeks postoperatively

*bAVM* brain arteriovenous malformation, *CT* computed tomography, *DS* digital subtraction, *NICU* neurointensive care unit, *SBP* systolic blood pressure

For postoperative treatment, patients are divided to three groups, based on the AVM characteristics and surgical complications (SMG and whether or not the AVM had ruptured preoperatively) (Table 1).

The patients in all three groups are taken back into surgery for reoperation if postoperative angiography shows that the AVM has not been completely extirpated. If, however, the computed tomography (CT) scan shows hemorrhage or edema, the patient is kept hypotensive and sedated under controlled ventilation. If the imaging shows that a non-AVM related artery is obstructed, the blood pressure is increased up to 130–150 mmHg.

Postoperatively antiepileptic medication is given to all patients with supratentorial AVMs. Furthermore, if embolized preoperatively, glucocorticoid (betamethasone or dexamethasone) is started. For prevention of deep venous thrombosis, antiembolic stockings or venous pumps are used for the first postoperative week instead of pharmacological treatment because of increased risk of hemorrhage.

According to the Helsinki protocol, good collaboration between neuroradiologist, neurosurgeon and neuroanesthesiologist is essential when deciding the postoperative treatment [20].

### Statistical Analysis

Statistical analysis was performed using IBM SPSS Statistics (version 24, IBM Corporation) program. Comparisons between variables were performed using Mann–Whitney *U* test, Pearson *χ*^2^, and Welch’s *t* test (variances tested with Levene’s test) as appropriate with SPSS. A statistical significance level of 5% was used for all analysis.

## Results

**Fig. 1:**
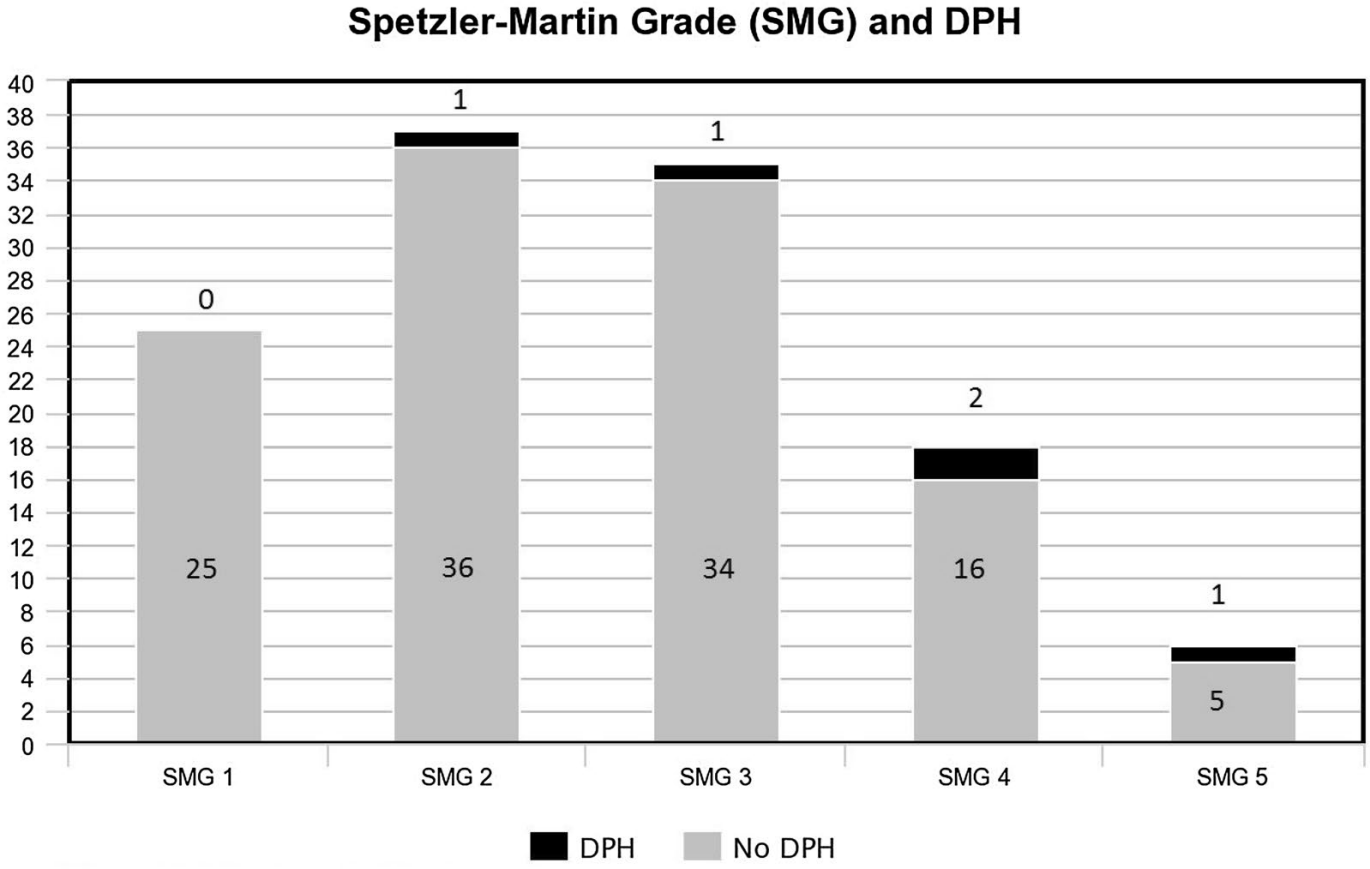
DPH by Spetzler–Martin grade

Five patients out of 121 (4.1%) were identified with DPH after neurosurgical treatment of bAVM. When divided according to SMG, the incidence for DPH increases from grade 1 to 5: 0%, 2.7%, 2.9%, 11% and 17% (Fig. 1), respectively, *p* = 0.043 (Table 2). Deep venous drainage was related to DPH (*p* = 0.003) (Table 2). No patient with purely cortical venous drainage suffered from DPH. Four patients who had DPH had both cortical and deep and one had purely deep venous drainage (Table 2). All the patients with DPH were male and over 18 years of age at the time of operation. Relationship between gender and DPH was statistically significant with *p* = 0.040 (Table 2).

**Table 2:** Characteristics of the study

				Was there delayed postoperative hemorrhage		
				No	Yes	p=
Spetzler–Martin	1	Sex	Female	13	0	
			Male	12	0	
			Total	25	0	
		All daily SBP targets achieved	No	10	0	
			Yes	15	0	
			Total	25	0	
	2	Sex	Female	14	0	
			Male	22	1	
			Total	36	1	
		All daily SBP targets achieved	No	20	1	
			Yes	16	0	
			Total	36	1	
	3	Sex	Female	17	0	
			Male	17	1	
			Total	34	1	
		All daily SBP targets achieved	No	20	0	
			Yes	14	1	
			Total	34	1	
	4	Sex	Female	6	0	
			Male	10	2	
			Total	16	2	
		All daily SBP targets achieved	No	8	1	
			Yes	8	1	
			Total	16	2	
	5	Sex	Female	4	0	
			Male	1	1	
			Total	5	1	
		All daily SBP targets achieved	No	1	1	
			Yes	4	0	
			Total	5	1	
	Total	Sex	Female	54	0	
			Male	62	5	
			Total	116	5	,040^a^
		All daily SBP targets achieved	No	59	3	
			Yes	57	2	
			Total	116	5	,689^a^
		Any daily SBP targets achieved	No	5	0	
			Yes	111	5	
			Total	116	5	,635^a^
		Spetzler–Martin		116	5	,043^b^
Mean age on admission (years) (SD)				42,26 (18,37)	46,53 (15,32)	,574^c^
Was embolization performed before surgical excision	No			77	3	
	Yes			36	1	
						,772^a^
Associated aneurysm, yes/no	No			80	3	
	Yes			26	1	
	Total			106	4	,983^a^
Venous drainage	Cortical			69	0	
	Deep			17	1	
	Cortical and deep			25	4	
	Total			111	5,00	,003^b^
Venous drainage	Cortical	Mean AVM size in mm (Angiography or CT largest diameter) (SD)		24 (14)		
	Deep	Mean AVM size in mm (Angiography or CT largest diameter) (SD)		18 (10)	16 (–)	
	Cortical and deep	Mean AVM size in mm (Angiography or CT largest diameter) (SD)		40 (21)	52 (25)	
Outcome at 2–4 mos (1. follow-up); GOS	Good recovery			53	1	
	Moderate disability			29	1	
	Severe disability			19	3	
	Vegetative state			0	0	
	Dead			6	0	
	Not known			9	0	
	Total			116	5	,278^b^

*AVM* arteriovenous malformation, *CT* computed tomography, *GOS* Glasgow Outcome Scale, *SBP* systolic blood pressure, *SD* standard deviation

^a^Pearson Chi-square

^b^Independent samples Mann–Whitney *U* test

^c^Welch’s *t* test

The median bAVM size in patients who did not have DPH was 20 mm (mean 26 mm), while in patients with DPH the median bAVM size was 35 mm (mean 45 mm) (Table 3). The postoperatively bled bAVMs were of 16, 27, 35, 65 and 80 mm in diameter. The bAVM size did not correlate with incidence of DPH, *p* = 0.076 (Table 3). Patients with DPH had higher average daily intravenous fluid intake (3256 ml) during NICU stay compared to patients without DPH (2272 ml), a 43% relative increase, *p* = 0.052 (Table 4). NICU stay was longer in patients with DPH (*p* = 0.022). There was no difference in GOS at 2–4 months after operation between patients with or without DPH, *p* = 0.278. Patients with DPH had higher median SMG (4) than patients without (2). Succession rate in achieving all set SBP targets was achieved with 49% of the patients and was similar between patients with or without DPH (*p* = 0.689). Only five patients failed SBP target every day and none of them had DPH (Table 2).

**Table 3:** AVM size and DPH

			AVM size in mm (Angiography or CT largest diameter)
Was there delayed postoperative hemorrhage	No	Mean (SD)	26 (17)
		Median (IR)	20 (23)
		Maximum	88
		Minimum	5
	Yes	Mean (SD)	45 (27)
		Median (IR)	35 (51)
		Maximum	80
		Minimum	16
	p=		,076^a^
	Total	Mean (SD)	27 (18)
		Median (IR)	20 (23)
		Maximum	88
		Minimum	5

*AVM* arteriovenous malformation, *CT* computed tomography, *IR* interquartile range, *SD* standard deviation

^a^Independent samples Mann–Whitney *U* test

**Table 4:** Daily IV fluid intake in milliliters, operating physician (A = experienced attending neurosurgeon) and average NICU unit stay length in days by Spetzler–Martin grade and incidence of DPH

				Was there delayed postoperative hemorrhage			
				No	Yes	p=	Total
Spetzler–Martin	1	Operating physician	A	19	0		19
			Other	6	0		6
			Total	25	0		25
		Mean daily IV fluid intake (ml) (SD)		1824,97 (1347,23)			1824,97 (1347,23)
		Mean NICU days (SD)		4 (3)			4 (3)
		Mean time in days between intubation and extubation (SD)		4 (8)			4 (8)
	2	Operating physician	A	28	1		29
			Other	8	0		8
			Total	36	1		37
		Mean daily IV fluid intake (ml) (SD)		2251,16 (1269,99)	4310,40		2317,59 (1302,27)
		Mean NICU days (SD)		5 (4)	22		5 (5)
		Mean time in days between intubation and extubation (SD)		3 (5)			3 (5)
	3	Operating physician	A	27	1		28
			Other	7	0		7
			Total	34	1		35
		Mean daily IV fluid intake (ml) (SD)		2639,47 (1098,67)	2499,00		2634,79 (700,31)
		Mean NICU days (SD)		5 (5)	11		6 (5)
		Mean time in days between intubation and extubation (SD)		5 (8)	1		5 (8)
	4	Operating physician	A	14	2		16
			Other	2	0		2
			Total	16	2		18
		Mean daily IV fluid intake (ml) (SD)		2244,79 (1370,89)	3168,15 (994,40)		2347,38 (1343,71)
		Mean NICU days (SD)		8 (7)	16		9 (7)
		Mean time in days between intubation and extubation (SD)		5 (8)	3		5 (8)
	5	Operating physician	A	5	1		6
			Other	0	0		0
			Total	5	1		6
		Mean daily IV fluid intake (ml) (SD)		2313,76 (1625,19)	3133,10		2450,32 (1491,60)
		Mean NICU days (SD)		6 (4)	11		7 (4)
		Mean time in days between intubation and extubation (SD)		3 (2)	8		4 (3)
	Total	Operating physician	A	93	5		98
			Other	23	0		23
			Total	116	5	,269^a^	121
		Mean daily IV fluid intake (ml) (SD)		2271,71 (1281,20)	3255,76 (822,22)	,052^b^	2317,69 (1277,93)
		Mean NICU days (SD)		5 (5)	15	,022^b^	6 (5)
		Mean time in days between intubation and extubation (SD)		4 (7)	4	,954^b^	4 (7)

*DPH* delayed postoperative hemorrhage, *IV* intravenous, *NICU* neurointensive care unit, *SD* standard deviation

^a^Pearson Chi-square

^b^Welch’s *t* test

### Case Studies of Patients with DPH

#### Case 1

Fifty-two-year-old male was included, a smoker with hypertension and alcoholism. Imaging after epileptic seizure revealed a previously unruptured bAVM of 35 mm diameter (SMG 4) at the left Sylvian fissure. Preoperative embolization was not feasible.

The patient was operated on via a frontotemporal craniotomy. Large anteromedial draining vein ruptured intraoperatively but was successfully coagulated. Both M2 branches were temporarily clipped. Good hemostasis was achieved, and postoperative angiography showed complete removal of the AVM.

First POD CT-scan showed some hemorrhage around the operative area. SBP target was kept under 120 mmHg until raised to 140 mmHg on the third POD. At the sixth POD, CT-scan showed large hematoma in the temporal lobe. The hematoma was evacuated. The suspected source of bleeding was a small artery adjacent to the operative area.

Next day, CT-scan showed a new hematoma. Angiography found the source to be a flow related M1 aneurysm which was operated on the same day. Tracheostomy was performed on the 12th POD with SBP target of under 150 mmHg. On the 22nd POD, the patient was sent to the neurosurgical ward, on the 34th to local hospital. GOS at 5 months was 3.

#### Case 2

Twenty-year-old male with psychiatric history was diagnosed with a preoperatively ruptured SMG 4 bAVM of 65 mm in the right temporal lobe after presenting with intracranial and intraventricular hemorrhage.

The operation was performed via a frontotemporal pterional craniotomy. Major (1350 ml) intraoperative bleeding occurred during nidus dissection. Bleeding continued until the complete removal of the nidus. Intraoperative angiography showed no residual AVM. Postoperative SBP target was 90–120 mmHg.

CT-angiogram on the first POD showed early filling of a cortical vein. CT-scan showed small subdural hematoma and operative site hematoma, which did not require evacuation. The patient was extubated on the 2nd POD. On the 9th POD, the patient was transferred to a regional hospital where he soon developed a severe headache. CT-scan showed epidural blood collection around craniotomy area. The patient was transferred back to the neurosurgical unit where 30 ml of blood was aspirated with a needle on arrival, easing the headache and then recraniotomy was performed to evacuate the epidural hematoma. A small cortical lesion was seen under the dura and promptly coagulated. Epidural drain was left for 48 h postoperatively. CT-scan showed significant residual hematoma and air around the operative area. On the 4th day after recraniotomy, the patient was sent back to the regional hospital. GOS at 3 months was 5.

#### Case 3

Fifty-two-year-old, otherwise, healthy male was diagnosed with a ruptured 16-mm left-sided temporo-occipital AVM of SMG 3 after an epileptic seizure. The AVM was embolized twice with Onyx 6 months before the operation with partial occlusion of the nidus. The operation was performed via parieto-occipital craniotomy.

Digital subtraction angiography (DSA) on first POD showed complete removal of the nidus. Retrograde filling was perceived from small branches near the AVM bed. On the 4th POD, the patient had right-sided convulsions, dysphasia and headache. CT-scan showed intraventricular hemorrhage from the AVM site and hydrocephalus. A right-sided ventriculostomy was inserted. On the 8th POD, meningitis was diagnosed, and antimicrobial treatment started. The ventriculostomy was removed on the 10th POD. On the 13th POD, the patient was sent back to his local university hospital for further recovery with GOS 4.

#### Case 4

A forty-seven-year-old man was diagnosed with a preoperatively ruptured large right-sided temporal AVM (diameter 80 mm, SMG 5). The AVM was first diagnosed and embolized after bleeding at the age of 20. Further treatment included embolization and proton therapy. After the patient started to suffer from severe headache, hallucinations and vertigo, CT-scan was performed showing a small intraventricular hemorrhage.

The surgery was done via right-sided temporal craniotomy. The AVM was attached to the dura at multiple sites. Multiple temporary clips were placed on the feeding arteries during the operation. Intraoperative angiography showed filling also from the posterior circulation. The AVM started filling quickly from deep basal arteries after it was thought to be dissected successfully. These were coagulated and the nidus removed. Some occipital residual AVM was visualized and removed. There was some hemorrhage during clip removal which was coagulated. Blood loss during surgery was 300 ml.

The postoperative SBP target was 100–130 mmHg. CT-scan and DSA showed no complications at 1st POD. On the 3rd POD, CT-scan showed blood in subdural space, resection cavity and subcutaneously. Cerebrospinal fluid (CSF) analysis on the 3rd POD showed potential infection and vancomycin and ceftazidime were started. The patient was extubated on the 4th POD, but reintubated due to worsening general status on the 5th POD. The SBP target was set to normotension on the 5th POD. The patient was extubated on the 7th POD. The patient was transferred to city hospital on the 29th POD. GOS at the first follow-up was 3. The patient initially developed right-sided ptosis which resolved by itself but was blind in his right eye at first follow-up visit due to uncertain etiology. The patient was deemed receptive for occupational and physiotherapy.

#### Case 5

Sixty-year-old otherwise healthy male was admitted to the hospital 9 days prior to surgery because of explosive headache. Similar but less intense headache had occurred 3 months earlier. Imaging studies revealed subarachnoid and intraventricular hemorrhage caused by a previously ruptured left-sided frontal bAVM (diameter 27 mm, SMG 2) fed by the anterior cerebral artery (ACA). Four days prior to surgery, the patient became disoriented due to hydrocephalus and a ventriculostomy was placed.

The operation was performed via a paramedian frontoparietal craniotomy. Hemosiderin was found confirming the earlier bleeding. Pericallosal arteries were temporarily clipped anteriorly to the nidus during AVM dissection, and the nidus was removed. Due to the fragility of feeding arteries, postoperative SBP target was less than 100 mmHg; however, during closure, there was a peak in SBP up to 160 mmHg. Immediate postoperative imaging showed removal of the AVM and hematoma anterior to the resection cavity.

On the 1st POD, CT-scan showed increase in the hematoma size. SBP target was raised on the 2nd POD to under 120 mmHg. On the 3rd POD, the size of patient’s left pupil varied, but CT-scan showed no change in the hematoma size. EEG showed a possible epilepsy focus. Right-sided ventriculostomy was inserted which produced 20 ml of bloody CSF with high pressure. By the 4th POD, the drain had produced 95 ml of liquor over 15 cm H_2_O valve pressure. CT-scan confirmed good placement of the drain and good demarcation on the infarcts on both ACA supply areas. SBP upper limit was raised to 130 mmHg and the following day to 140 mmHg.

On the 7th POD, the SBP target was raised to less than 150 mmHg and a tracheostomy was performed. On the 10th POD, the sedation was eased, and the patient resumed spontaneous breathing. On the 14th POD, the patient was still ventriculostomy dependent and transferred to his local university hospital. MRI showed no improvement, and best neurological response was flexion with left-sided hemiparesis. GOS at 18 months was 3.

## Discussion

Helsinki protocol for postoperative treatment of bAVMs was applied in all patients in this study. Five out of 121 patients (4.1%) developed DPH. Incidence of DPH in our study is in line with incidence of DPH (4.4% after protocol implementation) in an earlier larger study by Morgan et al. [11] in which patients also followed targeted albeit a more complicated postoperative treatment protocol. There are great variations in the reported incidences of DPH (2–21%), some of which may be explained by different definition of DPH between the studies [5–10].

The risk for developing DPH following surgical resection is considerable, yet the benefit from complete cure from future bAVM-related hemorrhage is noteworthy. The risk for DPH is significantly higher when the SMG of the bAVM is 3 or higher. Smaller bAVMs with low SMG have very negligible risk for developing DPH.

Our postoperative treatment protocol and the one described by Morgan et al. for bAVMs are similar. The main difference is the simplicity of our protocol making its implementation easier for treatment personnel with less chance for error-related adverse effects. The perioperative treatment protocol used by Morgan et al. [11, 14] has been described in detail in earlier studies.

The Helsinki protocol used for all the patients in this study focuses on patients’ blood pressure and intravenous fluid regimen. For large AVMs, SBP is kept low for several days avoiding any peaks in blood pressure. Protocol published by Morgan et al. uses both mean arterial blood pressure (MAP) and cerebral perfusion pressure (CPP) for hemodynamic control in patients with SMG 3–5. Their target is to keep MAP under 70 mmHg and CPP over 60 mmHg or at over 50 mmHg with barbiturate induced coma, which leaves a very small window for intracranial and blood pressure levels. Barbiturate induced coma was not used in our protocol at all. Instead, propofol and dexmedetomidine were used for sedation postoperatively. Propofol was gradually changed to dexmedetomidine before extubation. Our protocol divided the patients into three groups taking into account the SMG and surgical treatment, whereas our colleagues divided their patient population to three groups based on SMG and bAVM diameter. The use of glucocorticoids was more limited in our study. Only those patients who were preoperatively embolized were started on betamethasone depending on the clinician’s judgment, whereas our colleagues’ protocol started all patients preoperatively on dexamethasone.

Deciding a proper perioperative treatment protocol for each patient is a very important decision. The more complex protocol presented by Morgan et al. would seem to work quite well for patients at high risk for DPH (Spetzler Ponce class C in their study), but as shown in our study a simpler protocol might be as appropriate. Patients with small risk for developing DPH may not benefit from such interventional and invasive treatment. Moreover, a less aggressive treatment protocol may shorten the NICU stay and reduce treatment related adverse events such as infections and also the cost of the treatment [21, 22].

The average NICU stay length for patients with DPH was 15 days and 5 for those without (6 days for the whole cohort), whereas the only reported length of NICU stay by Morgan et al. was for Spetzler–Ponce class C bAVMs being minimum of 7 days.

The use of induced barbiturate coma as a postoperative treatment therapy is not part of our protocol but was of considerable importance to the protocol introduced by Morgan et al. [11]. Induced barbiturate coma effectively decreases intracranial pressure. It has, however, notable adverse effects, including negative long-term cognitive effects [23, 24].

Morgan et al. [11] speculated that neurosurgeons skill development was a limiting factor in their study. In our study, most of the patients were operated by the same neurosurgeon with vast experience in bAVM surgery already from the beginning of the study.

### Comparison of Patients Who Developed DPH with Those Who Did Not

All the patients who developed DPH were male. Although statistically significant, reason for that might purely be the low number of DPHs. Males often have higher blood pressure than females throughout their lives, and hypertension is a major risk factor for DPH [25, 26]. However, female gender is associated with increased risk for subsequent hemorrhage for non-treated AVMs [27]. Females might be more suited for surgical treatment, if their risk for DPH is lower and bAVM hemorrhage higher.

The average daily intravenous fluid intake during NICU was 43% higher with patients who had DPH with *p* = 0.052, which borders on statistical significance. This affirms our protocols focus on strict fluid control, although a larger study population would be needed to confirm the possible effect fluid load has on incidence of DPH.

All daily SBP targets were achieved with about half the patients. Low achievement rate is partly explained by the strict interpretation. All SBP targets were failed in only five patients, none of whom developed DPH.

### Discussion of the Presented Patient Case Studies with DPH

We thoroughly reviewed the patients who had DPH to find out if choosing another treatment protocol would have prevented DPH. Case 1 had DPH on 6th POD even though a strict blood pressure control had been followed. This reinforces the understanding that redistribution of blood flow exposes brain parenchyma to great pressure possibly leading to bleeding. Smoking, hypertension and alcoholism may have contributed to the outcome.

Major intraoperative hemorrhage and large bAVM increased the risk of developing DPH for the second case and led to a very strict postoperative blood pressure control. One might consider whether the epidural hematoma should have been evacuated earlier or not. Probably, the hematoma was more related to the craniotomy than the AVM itself. Nevertheless, the patient was young and had an excellent recovery regardless of DPH.

The third case developed DPH on 4th POD. The patient was otherwise healthy aside from his bAVM and had moderate disability on the GOS at first follow-up. His outcome might have been better had he had his bAVM surgically excised without preoperative partial embolization altering the hemodynamic profile of the nidus.

The fourth patient was evaluated to have a high risk of postoperative complications. Strict protocol was applied. Bleeding in surgical cavity was thought to be related to surgery itself as there was no intraparenchymal component. In review, we do not believe that by treating the patient differently, we could have improved the outcome. The surgical risk for such complicated and large bAVMs is very high.

The fifth case is the most interesting in relation to the treatment protocol. Although the SMG was 2 and the bAVM small, a strict postoperative protocol was applied due to the preoperative bleeding and intraoperative findings. The operation was uneventful aside from the one blood pressure spike to 160 mmHg during closure. This blood pressure spike was the presumed cause of the DPH that was visualized on postoperative imaging. The consequences of one blood pressure spike during the operation emphasize the need for careful blood pressure management throughout the perioperative period.

The five patients who developed DPH show that however hard we try to control the risk factors for DPH, we are unable to completely prevent them from happening. The pathophysiology of bAVMs brings with it an inherent risk for DPH. We have used fluid restriction and hypotension to try to control the hemodynamic stress resulting from removal of the bAVM. Hypervolemia leads to increased hydrostatic pressure in the fragile capillary bed surrounding the AVM and in our opinion might cause edema, but scarcely hemorrhage. We do not believe the benefits from an even stricter fluid regimen would outweigh the adverse effects, especially since the incidence of DPH in our series is already comparable to the previously published series with a more strict protocol.

### Limitations of the Study

The cohort size of the study was small and the incidence of DPH rare. With only five incidents of DPH out of 121 participants in the study high level of statistical power are not achieved.

As our study was retrospective, we have no control group. Further study is required to produce more rigorous evidence on postoperative bAVM treatment protocols. However, bAVMs are a very rare disease, and their most feared complication, DPH, an uncommon complication.

## Conclusion

DPH was associated with high SMG, which supports its use in deciding postoperative treatment, complex venous drainage pattern, male gender and high intravenous fluid intake. Helsinki protocol for postoperative treatment of bAVMs seems to perform equally well when compared to a more complicated protocol. The benefits of simpler protocols are multifarious such as making the implementation of the protocol easier, decreasing the chance for treatment personnel error and reducing the risk for treatment related adverse event. Further study with greater study size would be required to produce more rigorous evidence for postoperative treatment of microsurgically resected bAVMs; however, thus far Helsinki protocol seems to perform adequately.
